# Gestation changes sodium pump isoform expression, leading to changes in ouabain sensitivity, contractility, and intracellular calcium in rat uterus

**DOI:** 10.14814/phy2.13527

**Published:** 2017-12-06

**Authors:** Rachel V. Floyd, Ali Mobasheri, Susan Wray

**Affiliations:** ^1^ The Department of Molecular and Cellular Physiology University of Liverpool Liverpool United Kingdom; ^2^ Department of Veterinary Preclinical Sciences School of Veterinary Medicine Faculty of Health and Medical Sciences University of Surrey Guildford United Kingdom

**Keywords:** Gestation, immunohistochemistry, isoform, K^+^‐ATPase, myometrium, Na^+^, smooth muscle

## Abstract

Developmental and tissue‐specific differences in isoforms allow Na^+^, K^+^‐ATPase function to be tightly regulated, as they control sensitivity to ions and inhibitors. Uterine contraction relies on the activity of the Na^+^, K^+^ ATPase, which creates ionic gradients that drive excitation‐contraction coupling. It is unknown whether Na^+^, K^+^ ATPase isoforms are regulated throughout pregnancy or whether they have a direct role in modulating uterine contractility. We hypothesized that gestation‐dependent differential expression of isoforms would affect contractile responses to Na^+^, K^+^ ATPase *α* subunit inhibition with ouabain. Our aims were therefore: (1) to determine the gestation‐dependent expression of mRNA transcripts, protein abundance and tissue distribution of Na^+^, K^+^ ATPase isoforms in myometrium; (2) to investigate the functional effects of differential isoform expression via ouabain sensitivity; and (3) if changes in contractile responses can be explained by changes in intracellular [Ca^2+^]. Changes in abundance and distribution of the Na^+^, K^+^ ATPase *α*,* β* and FXYD1 and 2 isoforms, were studied in rat uterus from nonpregnant, and early, mid‐, and term gestation. All *α*,* β* subunit isoforms (1,2,3) and FXYD1 were detected but FXYD2 was absent. The *α*1 and *β*1 isoforms were unchanged throughout pregnancy, whereas *α*2 and *α*3 significant decreased at term while *β*2 and FXYD1 significantly increased from mid‐term onwards. These changes in expression correlated with increased functional sensitivity to ouabain, and parallel changes in intracellular Ca^2+^, measured with Indo‐1. In conclusion, gestation induces specific regulatory changes in expression of Na^+^, K^+^ ATPase isoforms in the uterus which influence contractility and may be related to the physiological requirements for successful pregnancy and delivery.

## Introduction

The excitability of human myometrium must be regulated to control contractile activity for successful pregnancy and parturition (Wray et al. 2003, 2015; Noble et al. 2009). While the pivotal role of intracellular [Ca^2+^] has been well‐studied (Floyd and Wray 2007), other ions including K^+^, Na^+^, and Cl^−^ are also known to be important (Khan et al. 2001; Jones et al. 2004; Seda et al. 2007; Tong et al. 2011). Either directly (Na^+^, K^+^) or indirectly (Ca^2+^, Cl^−^), the concentrations of these ions are dependent upon the Na^+^, K^+^‐ATPase, as it moves 3Na^+^ out of the cell with 2K^+^ entering and contributes to the negative cell membrane potential and the differential concentration gradients of sodium and potassium. The ion distributions across the cell membrane can be linked to the entry or efflux of other ions, including Ca^2+^, Cl^−^ and protons, all of which will affect myometrial excitability (Wray et al. 2015). In smooth muscle Moore et al. (1993) showed co‐localization of the Na^+^, K^+^ ATPase and Na‐Ca exchanger (NCX), and Blaustein's group; (Blaustein et al. 1992; Blaustein 1993; Golovina et al. 2003) further showed that intracellular [Na^+^] in the sub‐sarcolemmal space influences the activity of the NCX and sarcoplasmic reticulum (SR) Ca^2+^ content. As myometrial SR approaches the plasma membrane throughout the cell (Wray and Shmygol 2007) and NCX contributes about a third to Ca^2+^ efflux (Taggart and Wray 1997; Matthew et al. 2004), effects of the Na^+^, K^+^ ATPase on Ca^2+^ availability and contraction will occur. In addition, it has been reported that ouabain, an inhibitor of the Na^+^, K^+^ ATPase and endogenous cardiotonic steroids (glycosides), target the Na^+^, K^+^ ATPase and initiate IP_3_ Src signaling (Zhang et al. 2008; Aperia et al. 2016). Recently it has been suggested that for this ouabain‐dependent signaling, the Na^+^, K^+^ ATPase resides in the caveolar (lipid raft) domain of the membrane (Yosef et al. 2016). Caveolae are abundant in smooth muscle, including the uterus and thus this could be another way in which the Na^+^, K^+^ ATPase can influence contraction (Draeger et al. 2005; Noble et al. 2006). In addition, the effect of ouabain on Na^+^, K^+^ ATPase activity is modified by its isoform composition (see below), as they vary in sensitivity, and this in turn changes Ca signals, due to increasing intracellular [Na^+^] deceasing NCX activity, and consequently increasing Ca^2+^ in the myocytes.

The Na^+^, K^+^ ATPase is a P‐type ATPase first described by Skou (1957). It is composed of two *α* subunits, two *β* subunits and usually a third, FXYD subunit. Each of these subunits has multiple isoforms encoded by distinct genes (Shull and Lingrel 1987; Martin‐Vasallo et al. 1989; Lingrel et al. 1990). The expression of the isoforms is both tissue (Orlowski and Lingrel 1988b) and species specific (Zahler et al. 1993, 1996). The *α* subunit has four isoforms, *α*1‐*α*4, but *α*4 is specific to spermatozoa. The *α* subunit has binding sites for nucleotides, cations and the inhibitory glycosides and is responsible for the Na^+^, K^+^ ATPase enzymatic activity. Three *β* isoforms have been identified (*β*1‐*β*3), which are thought to modulate cation affinity, and the folding and trafficking of the Na^+^, K^+^ ATPase. To date seven FXYD isoforms have been identified and they lower substrate affinities or Vmax of the Na^+^, K^+^ ATPase (Geering 2005).

It is now appreciated that each subunit isoform has somewhat different properties, and therefore that the Na^+^, K^+^ ATPase will have site specific differences in its function, susceptibility to glycosides, and regulation of ion transport. It is considered that all these attributes of the Na^+^, K^+^ ATPase are varied to best suit the activity of the tissue it is functioning in. Given the uterus relies on the Na^+^, K^+^ ATPase for its rhythmic contractions but also has a fundamental shift from near quiescence throughout most of pregnancy, to hours of powerful, prolonged contractions during labor, it may be that variations in the isoform composition of the Na^+^, K^+^ ATPase play a role in supporting these changes in activity (Floyd et al. 2010a). Some insights into what these changes might be can be gathered from the literature in other cells or tissues.

The *α*1 and *β*1 subunits are ubiquitously present in tissues, and the *α*1 and *β*1 isozyme combination is considered the basic housekeeping form of the Na^+^, K^+^ ATPase that all cells require (Clausen et al. 2017). The isoforms of the *α* subunit show differences in K affinity; highest in *α*1, while *α*3 has relatively low Na affinity (Blanco 2005). A relative expression of *α*3 to *α*1 several‐fold higher in men than in women as judged from RNA levels has also been reported (Gaborit et al. 2010). Interestingly, it has been suggested that the *α*2 subunit changes in response to activity in skeletal muscle (Kravtsova et al. 2016). This, along with earlier reports suggesting that of all the isoforms, *α*2 is most concerned with modulating [Ca], and that in *α*2 heterozygous mice, skeletal muscles and vascular smooth muscles are hyper‐contractile, develop force faster and show greater sensitivity to receptor stimulation than in wild‐type animals (He et al. 2001; Shelly et al. 2004), suggests that changes in the *α*2 subunit with gestation may be anticipated. Changes in Na^+^, K^+^ ATPase isoforms with gestation are also pointed to by the work of Tsai and colleagues, reporting in rat uterus that estradiol causes a decrease in *α*3 expression and a subsequent decrease in contractility (Tsai et al. 2000). As mentioned above, the *α* subunit isoforms vary in resistance to the effects of ouabain, with rat *α*1 being particularly resistant. Different concentrations of ouabain can therefore be used as a way to interrogate and functionally separate the *α* isoforms in a tissue (Monteith and Blaustein 1998). Ouabain at different concentrations has been reported indirectly to increase Ca^2+^ signaling in myometrium but no measurements of intracellular Ca^2+^ were made, and the very high concentrations of ouabain used were not designed to allow the effects on the different isoforms to be tested (Ausina et al., 1996).

The *β* subunits of the Na^+^, K^+^ ATPase are considered to modulate its functions as well as acting as chaperones to ensure maturation, expression at tight junctions and contributing to cellular adhesion and polarity (Geering 2008). The different *β* isoforms differ in their degree of post‐translational modification, especially glycosylation. The myometrium undergoes repeated transient hypoxic episodes as the strength of contractions is sufficient to compress blood vessels (Harrison et al. 1994; Alotaibi et al. 2015). Thus, it is of interest that the *β*1 subunit responds to oxidative stress by glutathionylation (Rasmussen et al. 2010), while *β*2 with the strongest effects on Na^+^, K^+^ ATPase kinetics, (reducing the apparent K affinity and raising external Na affinity (Larsen et al. 2014), may be anticipated to increase with myometrial activity. FXYD isoforms and changes in them have not been studied for functional effects in any smooth muscle, as far as we are aware.

Thus, different subunit isoforms convey distinct properties to the Na^+^, K^+^ ATPase, from kinetic properties, membrane localization and trafficking, sensitivity to endogenous and applied Na^+^, K^+^ ATPase inhibitors, which will also have secondary effects, such as on Ca fluxes (Blanco and Mercer 1998; He et al. 2001; Horisberger and Kharoubi‐Hess 2002). As all of these aspects are likely to impact on excitation contraction by varying ionic gradients and potentials, the question arises as to which isoforms are expressed in the uterus and whether this expression is varied during gestation, and if it can have any functional effect on uterine contractility. There is little literature concerning the *α* and *β* isoform or FYXD expression in the myometrium (Esplin et al., 2003; Floyd et al. 2003) and no information about changes throughout pregnancy.

We have used a variety of techniques to test the hypothesis that there would be isoform‐specific changes with gestation in the myometrium and that these would affect the contractile and Ca responses of the myometrium to Na^+^, K^+^ ATPase inhibition with ouabain. Therefore, the aims of this work were: (1) to determine the mRNA transcripts and quantitative protein expressions and tissue distribution of Na^+^, K^+^ ATPase isoforms from rat myometrium; (2) to determine the effects of gestation on their expression and distribution; (3) to investigate the functional effects of differential isoform expression by testing ouabain sensitivity; and (4) if changes in contractile responses can be explained by changes in intracellular Ca^2+^ concentration.

## Materials and Methods

### Tissue

All studies were performed on female Sprague‐Dawley rats (Charles River, Kent UK). Uterine horns from nonpregnant rats (*n* = 4), early‐stage pregnant rats (10/11 days *n* = 4), mid‐stage pregnant rats (16/17 days *n* = 4) and late‐stage pregnant rats (21/22 days *n* = 4) were cleaned of adipose tissue and either used fresh for contractility studies, snap frozen in liquid nitrogen for western blot analysis or preserved in RNAlater for RT‐PCR studies. Tissues were fixed in neutral buffered formalin (NBF) for 24 h prior to paraffin embedding and preparation of custom designed tissue microarrays (TMAs) (Floyd et al. 2010a).

### SDS PAGE and western Blotting

Tissues were homogenized in the presence of protease inhibitor cocktail (Sigma, UK) and centrifuged at 10,000*g* for 10 min at 4°c to remove insoluble material. Protein concentration was determined using the BioRad Dc protein assay as directed by the manufacturer (BioRad, UK).

Extracted total proteins were separated by SDS‐PAGE on 12% gels for analysis of *α* and *β* subunits and 14% gels for FXYD‐ determination with 50 *μ*g protein loaded per lane against 5 *μ*L SeeBlue^®^ Plus2 Pre‐Stained Protein Standard (Invitrogen Ltd, UK) using previously described methods (Floyd et al. 2008). Primary antibodies were as follows: *α*620 1:100, *α*1 specific (a generous gift from Michael Kaplan), McHERED 1:2000, *α* 2 specific (a generous gift from Alicia McDonough, University of Southern California (Muller‐Ehmsen et al. 2001), MA3‐915 1:1500, *α* 3 specific (Affinity Bioreagents), SpET *β*1 1:1500, *β* 1 specific and SpET *β*2 1:1500, *β* 2 specific (both were generous gifts from Pablo Martín‐Vasallo, Universidad de La Laguna, Spain)(Gonzalez‐Martinez et al. 1994) and RNT*β*3 1:1500, *β* 3 specific (a generous gift from Dr Kathleen Sweadner, Harvard University). Positive and negative controls were included on each of the four repeat‐blots for each subunit to ensure a consistent, reproducible signal was generated.

### RT‐PCR

Total RNA was extracted from frozen rat tissues and quantified by spectrophotometry at 260 nmol/L. Reverse transcription was performed on equal amounts of template from each tissue group and template was assessed for integrity using intron spanning *β* actin primers. Amplification of cDNA templates was performed with species and isoform‐specific primer pairs using published primer sequences for *α*1‐3 and *β*1‐3 (Betts et al. 1997; Macphee et al. 2000) Na^+^, K^+^ ATPase, FXYD1 (Arystarkhova et al. 2007) and FXYD2a‐c (Jones et al. 2001) and transcripts were run on 1% agarose for analysis and sequencing.

### Immunohistochemistry

Tissue microarrays were constructed from formalin fixed, paraffin‐embedded rat tissues as described previously (Floyd et al. 2010a). Briefly, sections were de‐waxed in xylene and transferred through graded alcohols before antigen retrieval in boiling 10 mmol/L citrate buffer pH6. Endogenous peroxidase activity was blocked with 1% H_2_O_2_ in methanol before slides were washed in TBS/0.05% Tween 20 (TBST) and blocked in 5% BSA/TBST for 1 h at room temperature. Slides were washed with TBST and incubated with the following primary antibodies diluted in blocking solution overnight at 4°c: *α*6F neat supernatant, *α*1 specific (Developmental Studies Hybridoma Bank (Arystarkhova and Sweadner 1996), HERED 1:300, *α*2 specific and TED 1:300, *α*3 specific (both generous gifts from Thomas Pressley, Texas Tech University) (Pressley 1992), SpET *β*1 1:200, *β*1 specific and SpET *β*2 1:200, *β*2 specific (both generous gifts from Pablo Martín‐Vasallo, Universidad de La Laguna, Spain (Gonzalez‐Martinez et al. 1994), RNT*β*3 1:200, *β*3 specific (also Dr Kathleen Sweadner, Harvard University 3 (Arystarkhova and Sweadner 1997), *γ*a/b a and b specific, 1:200, (a generous gift from Steven Karlish, Biological Chemistry, Weizmann Institute of Science, Israel (Kuster et al. 2000) and Plm 1:100, phospholemman specific (also Steven Karlish, Weizmann Institute) (Crambert et al. 2002). Antibody‐antigen complexes were visualized with peroxidase‐conjugated EnVision™ polymer and DAB substrate (from DAKO, UK). Sections were counterstained in hematoxylin and mounted in DPX.

### Measurement of force and calcium

Myometrial tissue strips (3 × 1 mm) were dissected and loaded for 3 h at room temperature with a solution containing the ratiometric fluorophore Indo‐1 as described previously (Babiychuk et al. 2004). After the tissues had been incubated with the calcium indicator, strips were rinsed in physiological saline and transferred to a small (200 *μ*L) bath on the stage of an inverted microscope. The loaded tissues were excited with light at 340 nm and photomultipliers used to record the emitted light at 400 and 500 nm, and the ratio of these two emissions used to indicate changes in intracellular Ca. Tissues were fitted with aluminum clips, with one end attached to a force transducer (Grass FT03) and perfused with physiological saline at 32°C, to maximize Indo‐1 signaling and remain close to physiological parameters.

### Chemicals

All chemicals were purchased from Sigma (Dorset, UK). Antibodies were from DSHB (*α*6F) or otherwise kind gifts from Dr A. McDonough (McHERED), Dr T. Pressley (HERED and TED), Dr P. Martín‐Vasallo (SpET*β*1 and SpET*β*2), Dr K. Sweadner (RNT*β*3), Dr S. J. Karlish (*γ* a/b and plm). Protein quantification reagents were from Bio‐Rad, detection materials were purchased from Dako, Perbio Science (UK) or Amersham Biosciences (UK). Primers were synthesized by Proligo (France) all PCR substrates were purchased from Promega, Qiagen or Ambion (UK). Indo‐1 and Pleuronic^®^ F‐127 were from Molecular Probes (Oregon, USA). Ouabain was prepared in a stock solution of 10 mmol/L in distilled H_2_O and further diluted in physiological saline for use at 50 *μ*mol/L, 75 *μ*mol/L and 100 *μ*mol/L.

### Data presentation and statistical analysis

Western blot data was quantified using ImageJ software where data are expressed as a percentage of control *β* actin gels in terms of relative band intensity. Immunohistochemical analysis is referred to in terms of localization and distribution with inter‐tissue variability expressed in terms of density as TMA samples allow direct comparison to be made. Images were quantitatively evaluated and scored using previously published methods based on the spectral deconvolution method for DAB and hematoxylin, using IHC Profiler open source plugin for ImageJ. Five regions of interest were analyzed per image for areas delineated as smooth muscle, stroma, and epithelia at 40× magnification (Varghese et al. 2014).

Force/calcium data are given as mean and standard error of the mean (SEM), where ‘*n’* represents the number of samples, each taken from a different animal. Significance was tested, using ANOVA and where appropriate, Tukey's multiple comparison post hoc test where *P* values < 0.05 were accepted as significant. Data are expressed as percent changes in integral, (area under the curve, arbitrary units) for contractions and Ca changes and compared to preceding ten minutes’ control responses.

## Results

### Expression of mRNA transcripts encoding Na^+^, K^+^ ATPase *α* and *β* and FXYD2 isoforms in rat myometrium

**Figure 1:**
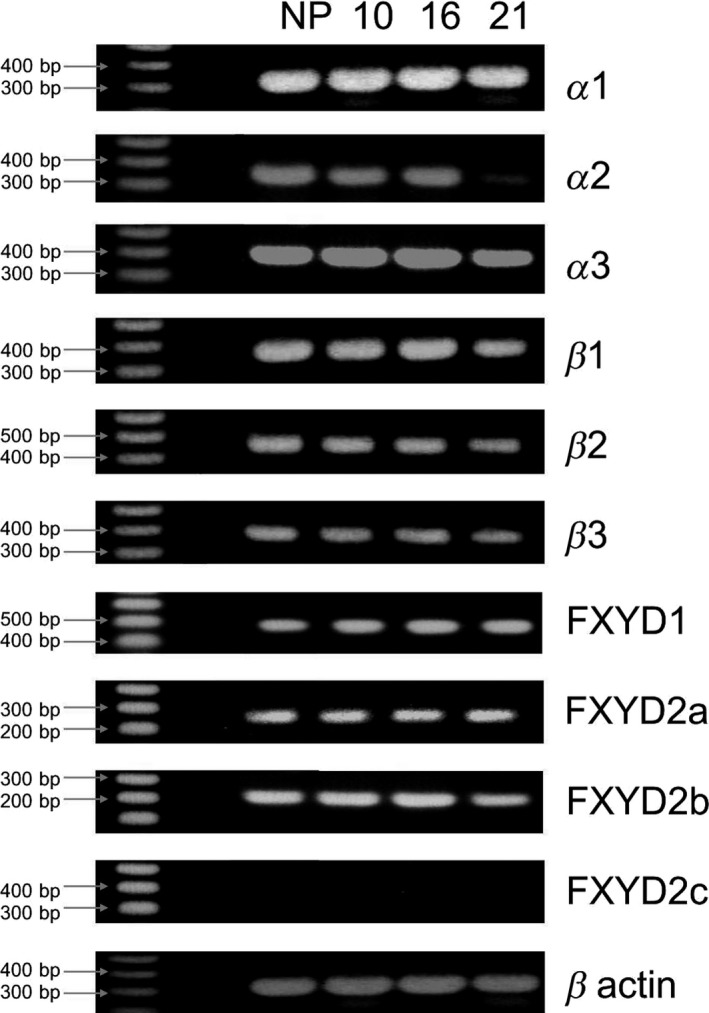
Analysis of mRNA transcripts corresponding to *α* 1‐3, *β* 1‐3 and FXYD2 a, b, and c isoforms of Na, K‐ATPase in uteri from nonpregnant (NP) rats and gestation days 10, 16, and 21 pregnant rats.

**Figure 2:**
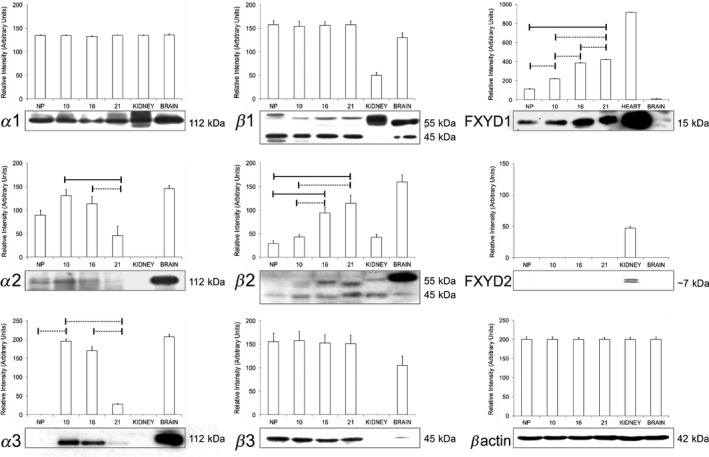
Quantitative Western blot analysis of Na, K‐ATPase *α*,* β* and FXYD isoform protein expression in uteri from non‐pregnant (NP) rats and gestation days 10, 16, and 21 in pregnant rats. Blots are expressed as a % of relative intensity of beta actin lane‐controls; error bars are SD of 4 technical replicates; where dotted line *P* < 0.05 filled line *P* < 0.001.

The expression of multiple isoforms of the Na^+^, K^+^ ATPase and FXYD1 and FXYD2 was studied in nonpregnant rat uterus and at different stages of pregnancy (days 10, 16, and 21). The typical expression pattern for each tissue group is shown in Figure 1. Amplicons of 336 bp encoding the *α*1 isoform, were consistently generated from each of the four groups of cDNA. The 335 bp transcripts encoding the *α*2 subunit were detected in all samples, similarly, detection of the 336 bp *α*3 isoform transcript was reliably reproducible in all groups. These data correlate with quantitative protein expression data shown in Figure 2. Amplicons corresponding to the *β*1, *β*2 and *β*3 genes (378 bp, 441 bp, and 384 bp, respectively), were generated with 100% frequency in all cDNA preparations from all groups studied. Amplicons corresponding to FXYD1 were detected in all samples. Detection of amplicons of 234 bp and 217 bp corresponding to the FXYD2a and 2b mRNA were consistently detected in samples from all groups with equal frequency. No evidence of FXYD2c mRNA was detected in any of the samples (bottom of Fig. 1). All repeat analysis on subsequent cDNA preparations produced identical results. Beta actin was consistently detected in all preparations from all gestational time points. The positive mRNA transcripts provided the basis for undertaking the investigation of protein expression and quantification.

### Expression of Na^+^, K^+^ ATPase *α* isoform proteins in rat uteri

Identification of changes in isoform expression during pregnancy through western blotting, allowed quantitative comparison to be made between protein samples. Figures are expressed as a percentage of the 45 kDa *β* actin control in units of relative intensity. The rat uterus (*n* = 4) displayed a pattern of distinct regulatory changes in Na^+^, K^+^ ATPase isoform expression throughout gestation as compared to nonpregnant animals of the same age. The 112 kDa *α*1 isoform (Fig. 2) was detected at all stages of gestation and also in the nonpregnant uterus. Expression in the uterus did not significantly change throughout the course of pregnancy, showing similar expression levels to control samples of kidney and brain. As gestation progressed, the expression of the 112 kDa *α*2 isoform decreased significantly by approximately 62% at term (22 days), after an initial significant 45% increase in intensity on the 10th day of pregnancy when compared to the nonpregnant uteri. Expression of *α*3 protein was significantly increased from extremely low nonpregnant values, at day 10 and 16 of gestation, before significantly decreasing (by 90%) in density toward term. There also appears to be no renal *α*3 but dense expression of the isoform in the brain which agrees with published literature (McDonough et al. 1994; Feraille et al. 1995).

### Expression of Na^+^, K^+^ ATPase *β* isoforms and FXYD2 proteins in rat uteri

The *β*1 isoform was detected by western blot at two different molecular weights that correspond to the variable state of glycosylation seen in most tissues that express this protein. The 56 kDa protein (Fig. 2) shows identical expression patterns to that of the 35 kDa unglycosylated form in all stages of pregnancy. The nonpregnant rat uterus and all stages of pregnancy show maintained levels of *β*1 protein. Expression of the *β*1 subunit was also present in both kidney and brain which agrees with previous studies (Feraille et al. 1995; Martin‐Vasallo et al. 1997, 2000). The 56 kDa *β*1 subunit is normally found in brain and skeletal muscle, not renal tissues so the pattern of expression seen in the control tissues is supported by current literature. Western blots show that the *β*2 isoform is more abundantly expressed at 16 days of gestation and at term in the rat uterus. Our studies show that the 56 kDa *β*3 isoform is consistently expressed in the rat uterus both in the virgin and pregnant states, with comparatively lower levels seen in kidney and brain as expected (Arystarkhova and Sweadner 1997).

Detection of FXYD2 using a pan‐*γ* specific antibody reveals a characteristic doublet on SDS PAGE gels at 6 kDa (Figure 3.3) (Kuster et al. 2000). This data shows that *γ*a/b is not detected in rat uterus at any stage of gestation or in adult rat brain by Western blot but is abundant in the kidney, while FXYD1 is ubiquitously expressed and rises significantly toward term.

### Distribution of Na^+^, K^+^ ATPase *α* isoforms in rat uterus

**Figure 3:**
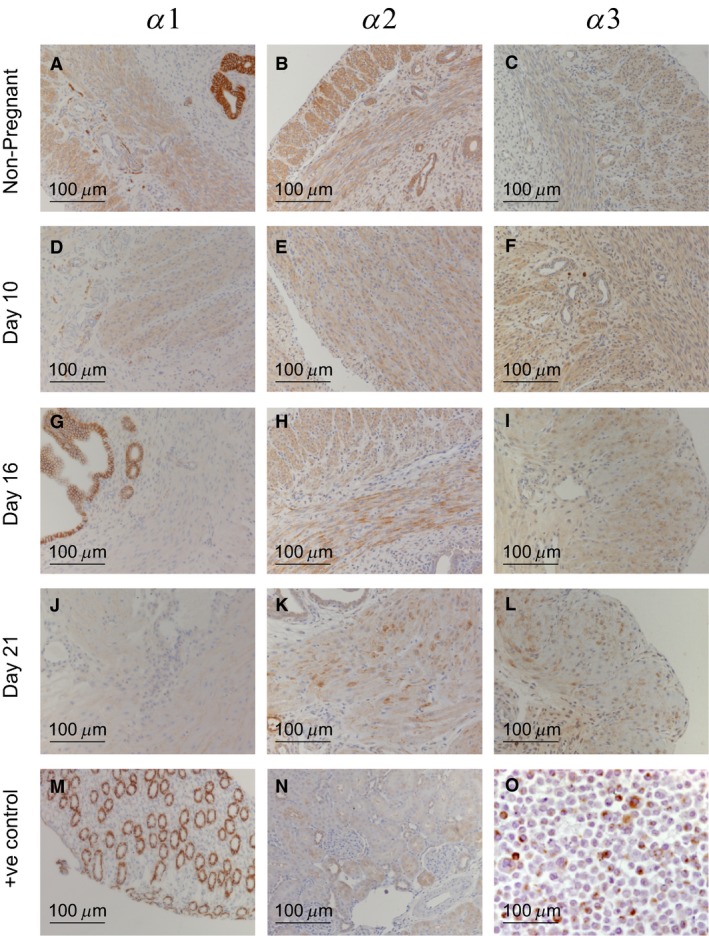
Distribution of Na, K‐ATPase *α* 1‐3 isoforms in uteri from non‐pregnant (NP) rats and gestation days 10, 16, and 21 pregnant rats, was determined by immunohistochemistry using isoform specific antibodies on tissue microarrays.

The localization of isoforms of the Na, K‐ATPase was studied in fixed tissues from nonpregnant, day 10, 16, and 21 of pregnancy (Fig. 3). Quantitative evaluation scores are included in Figure S1 for smooth muscle (A) and epithelial cell layers (B). Expression of the *α*1 isoform was determined using a well characterized specific monoclonal antibody *α*6F which binds to an N‐terminal region of the protein between residues 27‐55 (DSHB literature). Expression of *α*1 protein was most dense in the basolateral membranes of the secretory epithelia lining the endometrium (Fig. 3, panels A, D, G and J and Figure S1 A and B). Restricted pockets of sparse *α*1 expression were observed in the smooth muscle layers but was absent from within the endometrium.

Widespread expression of *α*2 protein was seen in all uterine samples analyzed (Fig. 3, B, E, H and K and Figure S1 A and B). Distinct localization was apparent in the longitudinal and circular smooth muscles of all samples, with little cross‐reactivity in the connective tissues of the myometrium and endometrium. Furthermore, epithelia lining secretory glands within the endometrium showed particularly strong expression of *α*2 in basolateral membranes (Panel B). Of particular interest is the distinct decrease in the intensity of immunostaining seen in the smooth muscle of samples taken from rats at term gestation (Panel K). This data correlates well with the significant decrease in *α*2 protein (Fig. 2) at this late stage of pregnancy (21 days). Immunohistochemical analysis of uteri from all groups of animals demonstrated very diffuse expression of *α*3 protein across all anatomical regions (Fig. 3, panels C, F, I, and L and Figure S1 A and B). Expression of the *α*3 isoform in smooth muscle was sparse across both the longitudinal and circular regions with little variation seen between the 5 groups, aside from being particularly faint on day 21, (Panel L). Expression of *α*3 in the luminal and secretory epithelia was low with no polarization in expression as seen in the *α*1 and *α*2 localization. No expression was observed in the connective tissue of the endometrium and myometrium.

### Distribution of Na^+^, K^+^ ATPase *β* isoforms in rat uterus

**Figure 4:**
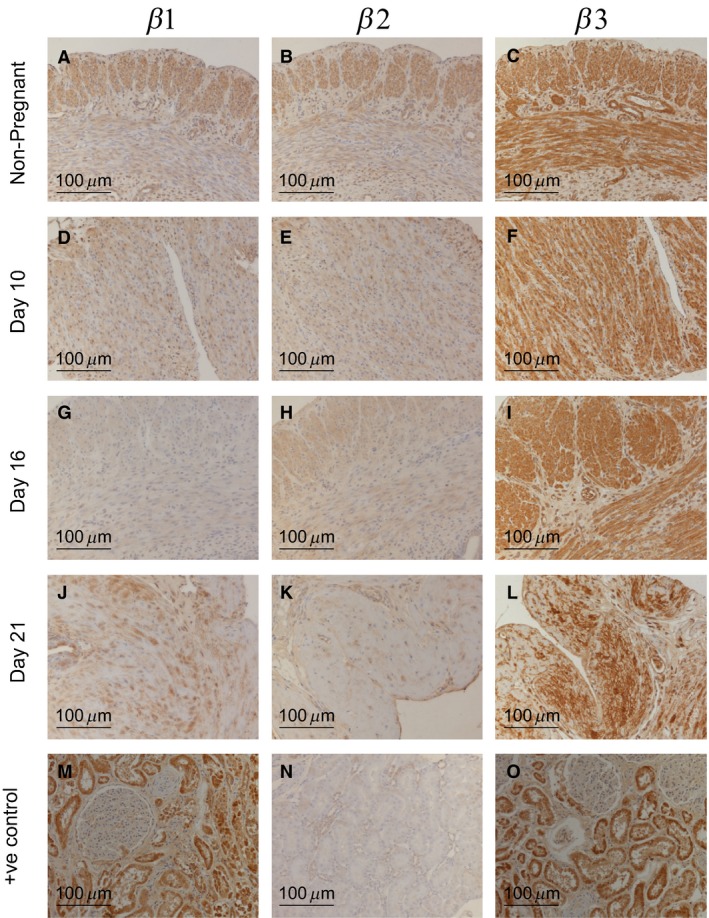
Distribution of Na, K‐ATPase *β* 1‐3 isoforms in uteri from nonpregnant (NP) rats and gestation days 10, 16, and 21 pregnant rats, was determined by immunohistochemistry using isoform specific antibodies on tissue microarrays.

The distribution of *β* isoforms of the Na^+^, K^+^ ATPase showed much less variability than that seen in the *α* subunit isoforms in the same tissues. What becomes immediately apparent is the widespread expression of both *β*1 and *β*2 isoforms in most of the tissue groups analyzed (Fig. 4). The most abundant expression is localized in the smooth muscle bundles, this is clearly most prominent in the nonpregnant samples (Fig. 4, A and B and Figure S1 A and B). This is primarily due to the nonpregnant smooth muscle myocytes being relatively compact compared to the pregnant samples which show a marked increase in cell surface area and therefore give the appearance of more diffuse staining. Furthermore, the prevalent expression of *β*1 protein at all stages of gestation relates well to our previous western blot and mRNA analysis showing ubiquitous expression in all samples (Figs. 1 and 2). In contrast, the immunohistochemical analysis does not detect any specific decrease in *β*2 expression in nonpregnant and 10 day pregnant tissues (Panels B and E).

The abundant nature of the immunostaining of the *β*3 isoform suggests that this may be the predominant *β* subunit present in uterine tissue as the binding specificity of this antibody in rat tissue is well documented (Arystarkhova and Sweadner 1997; Arteaga et al. 2004). Biochemical data also confirms that this isoform is present in all uterine samples at uniform levels (Fig. 2).

### Distribution of Na^+^, K^+^ ATPase FXYD1 and FXYD2 in rat uterus

**Figure 5:**
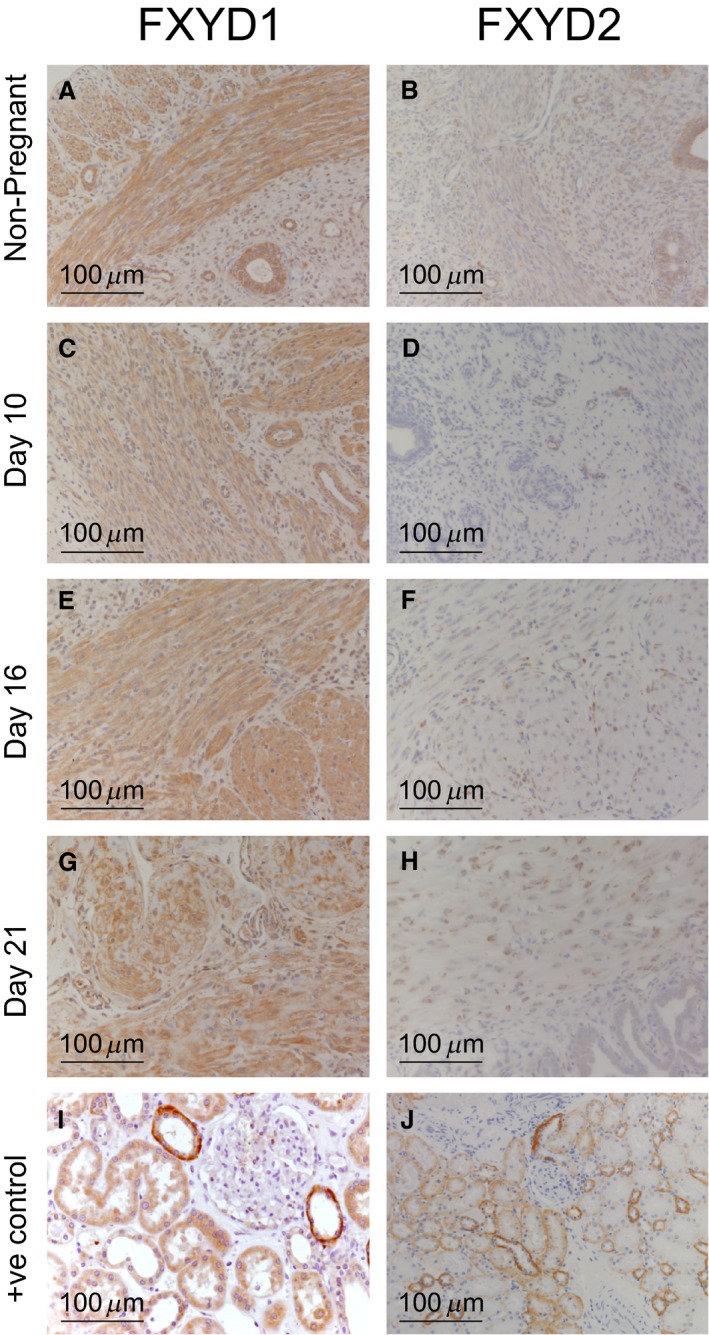
Distribution of FXYD1 & FXYD2 isoforms in uteri from nonpregnant (NP) rats and gestation days 10, 16, and 21 pregnant rats, was determined by immunohistochemistry using isoform specific antibodies on tissue microarrays.

Immunohistochemical analysis of the FXYD1 and 2 proteins was also performed on the rat tissue microarrays (Fig. 5 and Figure S1 A and B). Expression of FXYD1 was found to be widespread in all samples tested with no specific variation in abundance or localization. Conversely, very sparse immunolocalization corresponding to FXYD2 expression was seen in the smooth muscle bundles and secretory epithelia of all samples (Fig. 5 and Figure S1 A and B). No further expression of FXYD2 protein was seen in any other regions of these samples (Fig. 5 B, D F and H). Again, this is in good correspondence with the western data (Fig. 2) which failed to detect protein in all stages of gestation.

### Effects of Ouabain on contractility and intracellular Ca in NP, 10, 16, and 21 day pregnant rats

Contractile responses were analyzed by measuring the percentage change in the integral of contractions compared to a similar control period where tissues were perfused with physiological saline alone. The ouabain concentrations were chosen to differentially inhibit the *α* subunits (*α* 1 more resistant than *α* 2 which in turn is more resistant than *α* 3) (Jewell and Lingrel 1991) and contractile responses were investigated at the four stages of gestation. The lowest concentration at which any significant response was seen in both nonpregnant and term pregnant tissues was 50 *μ*mol/L (data from lower concentrations not shown).

The most marked and consistent effect on contractility was on frequency, calculated as number of contractions in a 10‐minute period. All samples, at all gestational stages, showed an increase in the frequency of contractions when exposed to 50, 75 or 100 *μ*mol/L ouabain.

**Figure 6:**
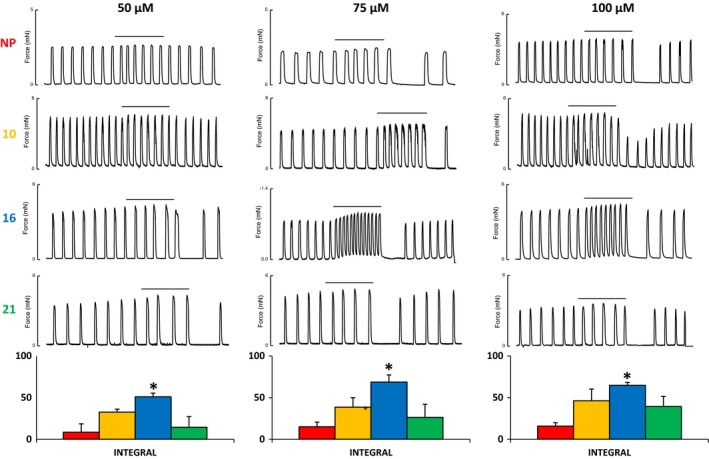
Dose‐dependent inhibition of alpha subunit isoforms of Na, K‐ATPase with 50 *μ*mol/L, 75 *μ*mol/L and 100 *μ*mol/L ouabain in myometrial strips from nonpregnant (NP) rats and gestation days 10, 16, and 21 pregnant rats. Data are expressed as % of 10‐minute control period immediately preceding ouabain exposure where * denotes *P* < 0.05.

Figure 6 shows representative force recordings from NP, 10, 16 and 21 day pregnant rats for each of the three concentrations of ouabain studied, and the bottom panel gives the mean concentration for each dose (*n *=* *4). It can be seen that the effects on contractility are dependent on the concentration of ouabain, with the largest difference occurring between 50 and 75 *μ*mol/L. The contractions of the uterus remained rhythmic and not tonic at all stages of gestation and with each ouabain concentration. The data also showed that the contractile activity in nonpregnant and term pregnant myometrium were consistently less affected by ouabain at each concentration, than the days 10 and 16 samples. As shown in the mean data the responses at day 16 were statistically larger than those at other gestations. These data are consistent with the protein data showing *α* subunits 2 and 3 being significantly more highly expressed at days 10 and 16 compared to nonpregnant and term myometrium, with these functional differences being more apparent at the lower ouabain concentrations.

**Figure 7:**
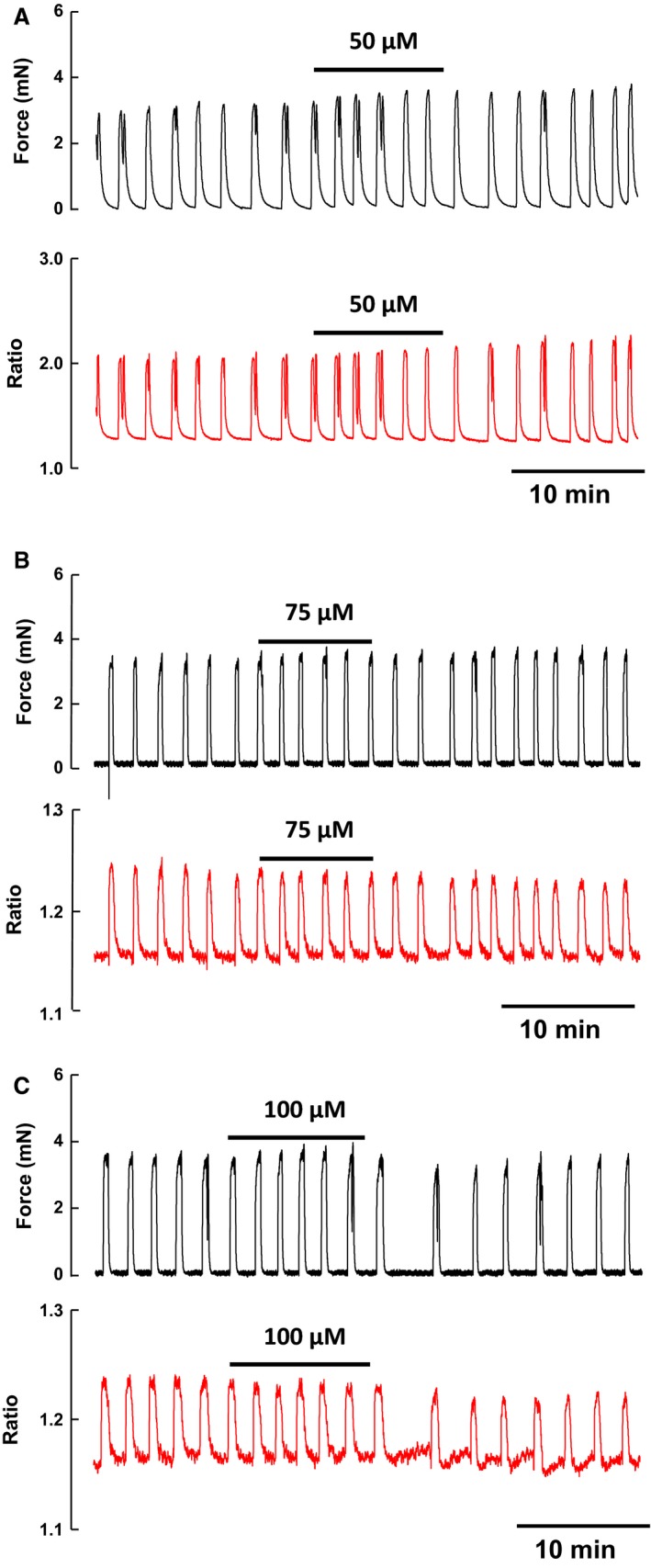
Relationship between force and calcium during ouabain inhibition of alpha subunit isoforms of Na, K‐ATPase with 50 *μ*mol/L (A), 75 *μ*mol/L (B), and 100 *μ*mol/L (C) ouabain in myometrial strips from nonpregnant (NP) rat.

Figure 7 shows typical traces from simultaneously recorded contractions and intracellular Ca (from Indo‐1 fluorescence) in nonpregnant rats. It can be seen that even at the highest ouabain concentrations there was no increase in basal Ca levels, consistent with there being no increase in basal force in these traces. The increases in active force were mirrored in the calcium transients in all experiments (*n* = 3).

## Discussion

The aims of this study were to determine the expression and distribution of the *α* and *β* isoforms of the Na^+^, K^+^ ATPase and the regulatory FXYD proteins in rat myometrium at different stages of pregnancy, in order to better understand how the Na^+^, K^+^ ATPase may affect uterine function. These data are the first to examine the distribution of both *α* and *β* Na^+^, K^+^ ATPase isoforms in uterine tissue from nonpregnant rats and throughout gestation. It is now appreciated that the Na^+^, K^+^ ATPase activity is modified by the expression of different isoforms of its molecular subunits, and so a determination of their expression and isozyme combination in the myometrium will increase our understanding of how the Na^+^, K^+^ ATPase will contribute to excitation‐contraction coupling during different stages of pregnancy. We show using biochemical, immunohistochemical and mRNA analyses that all three isoforms of both *α* and *β* subunits are expressed, along with FXYD1 in the myometrium. Interestingly three of these isoforms, *α*2, *α*3, and *β*2 change their expression with gestational changes suggesting that they are functionally regulated. The housekeeping isoforms *α*1 and *β*1 (Beguin et al. 2002), and *β*3 remained consistently expressed throughout all stages of pregnancy and in the nonpregnant myometrium. Detection of the *β*3 isoform was strong in all samples, in agreement with previous data suggesting that this isoform is widespread in its distribution (Arystarkhova and Sweadner 1997); it appeared to be the dominant *β* isoform of the myometrium. We also found that changes in sensitivity to ouabain occurred as a result of pregnancy and in a dose‐dependent manner. These changes are likely related to the aforementioned changes in isoform expression with gestation, as the subunits vary in their sensitivity (Jewell and Lingrel 1991). We also examined how inhibition of the Na^+^, K^+^ ATPase with low, medium and high ouabain concentrations affected Ca within the cell and found changes in the Ca transients, especially their frequency, underlay the force changes, but found little or no change in basal Ca or change from rhythmic to tonic contraction, as had been suggested from studies in other tissues (Hartford et al. 2004; Dostanic‐Larson et al. 2005, 2006; Saini‐Chohan et al. 2010).

Our study used a range of techniques to help build the fullest picture of the Na^+^, K^+^ ATPase isoforms in myometrium; mRNA transcripts, western blotting, immunohistochemistry, contractility assays and measurement of intracellular Ca. These different approaches provided a consistent pattern of data increasing confidence in the conclusions draw from the study. Western blotting from tissue homogenates will of course detect cells other than uterine myocytes, but it is estimated that the smooth muscle cells contribute >95% of the cellular content of the uterus (Wynn 1977). In addition, western blotting is not as sensitive as immunohistochemistry. Our data suggests that all the gene products for *α* and *β* subunits in mRNA are transcribed into protein and detectable by immunohistochemistry, but in nonpregnant samples western blots failed to detect *α*3 and *β*2. In addition, western blots showed all three *β* isoforms but the predominance of *β*3 was a clear finding on all the tissue sections, using immunohistochemistry. Similarly, the findings concerning Na^+^, K^+^ ATPase inhibition were consistent when force alone was measured and when intracellular Ca was simultaneously with force; the frequency of contractions, and Ca transients, increased. These consistent outcomes allowed us to minimize the number of rats required, in keeping with ARRIVE guidelines, although, while clearly able to detect the major changes occurring in contractions, the reduced statistical power may have led to us missing some smaller effects as being significant, due to the biological variations affecting force.

The impetus for this investigation came from previous studies showing a difference in *α* isoform expression between nonpregnant and pregnant human myometrium (Floyd et al. 2010b). These data pointed to a dynamic regulation of Na^+^, K^+^ ATPase expression, but the unavailability of samples throughout human gestation, precluded an investigation of this. The rat myometrium, which behaves very similarly to the human myometrium for excitation‐contraction coupling (Wray et al. 2015) provides a good model for testing the hypothesis that gestation will produce specific changes in Na^+^, K^+^ ATPase isoforms expression and distribution. That changes might be anticipated with the alterations in membrane potential, ion channel and exchanger expression and regulation, and increased contractility, necessary between early to late gestation, underlay our hypotheses. It has also been reported that the excitatory effect of PGE and PGF is followed by hyperpolarization due to Na^+^, K^+^ ATPase stimulation and that this decreases the frequency of subsequent contractions (Parkington et al. 1999). The amplitude of the hyperpolarization decreases during labour, allowing contraction frequency to increase. Its persistence at this time ensures complete relaxation between each single robust contraction, preventing spasm of the uterus that would restrict blood flow to the fetus during delivery.

Changes in Na^+^, K^+^ ATPase activity were also considered due to literature showing that sex steroids can influence Na^+^, K^+^ ATPase activity and isoform expression in cardiac and uterine tissue; estradiol has a direct stimulatory on the cardiac Na^+^, K^+^ ATPase via K (Dzurba et al. 1997), due to increased expression, and phosphorylation of the *α*1 subunit (Obradovic et al. 2014). Furthermore, previous studies suggest that changes in *α*3 isoform expression can have functional consequences in uterine smooth muscle, where decreased expression was correlated with reduced contractility in estradiol‐treated rats (Tsai et al. 2000). Progesterone can upregulate the *β*1 isoform in mouse uterus (Deng et al. 2013), and *α* isoforms in rat uterus (Chinigarzadeh et al. 2015), with these same authors also reporting that estrogen had the opposite effect and might lower reabsorption of uterine fluid Na. Although the Na^+^, K^+^ ATPase is expressed in other smooth muscle cells, these studies are not extensive or detailed (Burke et al. 1991; Mobasheri et al. 2003a,b; Shelly et al., 2004; Dostanic et al. 2005; Baker Bechmann et al. 2016), so it is not possible to extrapolate from them to the uterus. The Na^+^, K^+^ ATPase current has been directly measured in smooth muscle cells from mesenteric artery (Nakamura et al. 1999), and it has also been reported that the smooth muscles have fewer Na^+^, K^+^‐ATPases than striated muscles, which also makes them harder to study (Allen et al. 1991). These data and conclusions were largely gathered from vascular smooth muscles and were not focused on the different subunit isoforms. The uterus, especially at term is much more muscular and active than any other smooth muscle, with cells up to 0.5 mm long and large (nA) L‐type Ca currents, and so may be expected to be closer to striated muscle than other smooth muscles in terms of activity and ionic demands, (Burke et al. 1991; Shmigol et al. 1998). Burke et al. (1991) in colonic smooth muscle found that Na^+^, K^+^ ATPase isoenzymes were differentially expressed in electrically dissimilar regions of the muscle (Burke et al. 1991).

The constant expression of *α*1 and *β*1 and detection in all myometrial tissues underlines their primary role in maintaining intracellular Na and K levels. Given the fundamental and constant requirement of the myometrium to use electrical and chemical gradients, set up by the Na^+^, K^+^ ATPase, for its rhythmic activity, these findings underlie how important these subunits are to function. The functional effects of inhibiting *α*1 are discussed later. These findings concerning the *α*1 and *β*1 subunits are consistent with findings in other electrically excitable tissues, especially the heart, where the constant expression of the *α*1 subunit in hearts from all mammals examined, has been related to its constant requirement to maintain ionic gradients as it constantly beats (Orlowski and Lingrel 1988a; Zahler et al. 1993, 1996). They differ, however, from the findings on peri‐implanatation mouse uterus where *β*1 was upregulated by progesterone (Deng et al. 2013), but this may reflect activity in endometrium and glands at this special period in development, and not the myometrium.

We also found the *β*3 isoform to be unchanged with gestation. Although not extensively studied in intact preparations, this isoform is thought to influence ion transport by modifying ionic affinities (Jaisser et al. 1994; Hilbers et al. 2016). Our data would point to these being essential properties for the Na^+^, K^+^ ATPase expressed in myometrium at all stages of pregnancy.

Alpha 2 and 3 were both found to be increased significantly (especially *α*3) in early‐ and mid‐ pregnancy and decreased significantly at term. This could be related to the need to maintain the myometrial membrane in a more hyperpolarized state and where Ca transients decreased throughout pregnancy until parturition. The localization of *α*2 to the plasma membrane juxtaposed to the SR, is proposed to have a significant role in regulating Ca via the NCX in smooth muscle, as microdomains of Na are formed (Juhaszova and Blaustein 1997). Changes in expression of the *α*2 isoform have been shown to have critical function in both cardiac and neuronal activity (James et al. 1999; Muller‐Ehmsen et al. 2002; Moseley et al. 2003). In work on neonatal rats cerebellum, the ongoing hyperpolarization associated with development has been directly related to stimulation of the Na^+^, K^+^ ATPase and selective upregulation of *α*3 (Biser et al. 2000). Although we can find no direct measurements on how these isoform changes affect membrane potential in the uterus or any other smooth muscle, it is tempting to speculate the changes contribute to the mechanisms maintaining resting membrane potential, but further studies are required. The decrease in these isoforms at term is also consistent with the known decrease in membrane polarization at this time, as excitability is increased. In vascular smooth muscle, chronically reduced *α*2 in genetically modified mice lead to increased vascular resistance and blood pressure, as Na^+^, K^+^ ATPase activity was reduced, leading to increased Ca via entry on NCX (Zhang et al. 2005). These findings are also consistent with our suggestion that the increased *α*2 expression in early and midterm myometrium may contribute to uterine relaxation. Finally, Maxwell and colleagues reported decreased *α*2 protein expression in pre‐eclamptic women's myometria (Maxwell et al. 1998). Preeclampsia is marked by elevated blood pressure and constriction of vessels, our finding of increased *α*2 expression in mid‐pregnancy is again consistent with it contributing to myometrial relaxation at this time.

The only *β* isoform to change was *β*2 and this was the only isoform to increase toward term. It has been suggested that this subunit isoform is important for membrane trafficking and caveolae localization. It may therefore be that this is used by the myometrium to increase the drive on contractility near term. As mentioned earlier, caveolae are present in the myometrium in abundance and affect signaling in phasic muscles (Babiychuk et al. 2004). There is, however, little known about which signaling pathways in any smooth muscle may be linked directly to the Na^+^, K^+^ ATPase acting as a receptor for endogenous cardiotonic steroids (Xie and Askari 2002; Wasserstrom and Aistrup 2005). We found that FXYD1 is the primary isoform expressed in all myometrial samples. It is likely that the FXYD2a and b gene is expressed in all uteri, as shown by the mRNA data, but may not be translated (unless needed functionally).

The physiological studies presented here offer evidence of functional changes occurring in the uteri of nonpregnant rats and those at different stages of gestation. The three *α*‐subunit isoforms, have been characterized, with, low (mmol/L), high (*μ*mol/L) and very high (nmol/L), affinities for ouabain in the order *α*1, 2 then 3, (Blaustein 1993; Blaustein et al. 2002). Our finding that the myometrium showed increased sensitivity to inhibition of the Na^+^, K^+^ ATPase with ouabain at days 10 and 16 day of pregnancy, correlates with increases in expression of ouabain sensitive isoforms of the Na^+^, K^+^ ATPase, that is, *α*2 and *α*3, at these stages of gestation. These data also are consistent with our biochemical and immunohistochemical findings. In the myometrium, we found that active force and Ca transients significantly increase with ouabain. The frequency of contractions and transients were the most obvious effects of ouabain, suggesting that the membrane was becoming more depolarized. We did not observe tonic contraction or rise in basal Ca. Data from a previous study on rat myometrial preparations showed that 100–300 *μ*mol/L ouabain induces a significant increase in rhythmic contractions and no increase in resting force (Ausina et al. 1996). These findings are in broad agreement with ours. However, if, as functional studies suggest (Monteith and Blaustein 1998), that 100 *μ*mol/L ouabain is sufficient to inhibit even the highly resistant rat *α*1 isoform, the previous study could not shed light on differ isoform contributions to these effects, and no gestational studies were made and Ca was not measured. Interestingly it has been observed that high circulating levels of endogenous ouabain are found during human pregnancy (Vakkuri et al. 2000) and postnatally (di Bartolo et al. 1995).

In summary, the different Na^+^, K^+^ ATPase subunit expression that we have found with gestation, allows its activity to be optimized to its role in the myometrium. These findings agree with and extend findings from earlier, preliminary studies reporting isoform switching in pregnancy in rat and human myometrium (Maxwell et al., 1998; Esplin et al. 2003; Floyd et al. 2010b). This is because trafficking, membrane domain, posttranslational modifications and ionic affinities and susceptibility to glycosides can all be changed via the isoforms expressed. We have also found functional difference, especially on contraction and Ca transient frequency, to Na^+^, K^+^ ATPase inhibition, which are gestation dependent. There remains much work to be done on directly linking these changes to problems of uterine function, such as preterm birth and dysfunctional labors, but findings in preeclampsia and hypertension in pregnancy, suggest this would be worthwhile.

## Conflict of Interest

None declared.

## Data Accessibility

## Supporting information




**Figure S1.** Quantitative evaluation of Na, K ATPase isoform immunoreactivity in (A) smooth muscle and (B) epithelial cell layers using spectral deconvolution.
