# Supplementary material for: Gestation changes sodium pump isoform expression, leading to changes in ouabain sensitivity, contractility, and intracellular calcium in rat uterus
Source: Physiol Rep. 2017 Dec 6;5(23):e13527. doi: 10.14814/phy2.13527 (PMC5727280; doi:10.14814/phy2.13527)
Supplement: Supplementary file 1 — Figure S1. Quantitative evaluation of Na, K ATPase isoform immunoreactivity in (A) smooth muscle and (B) epithelial cell layers using spectral deconvolution. [file PHY2-5-e13527-s001.pdf]

A

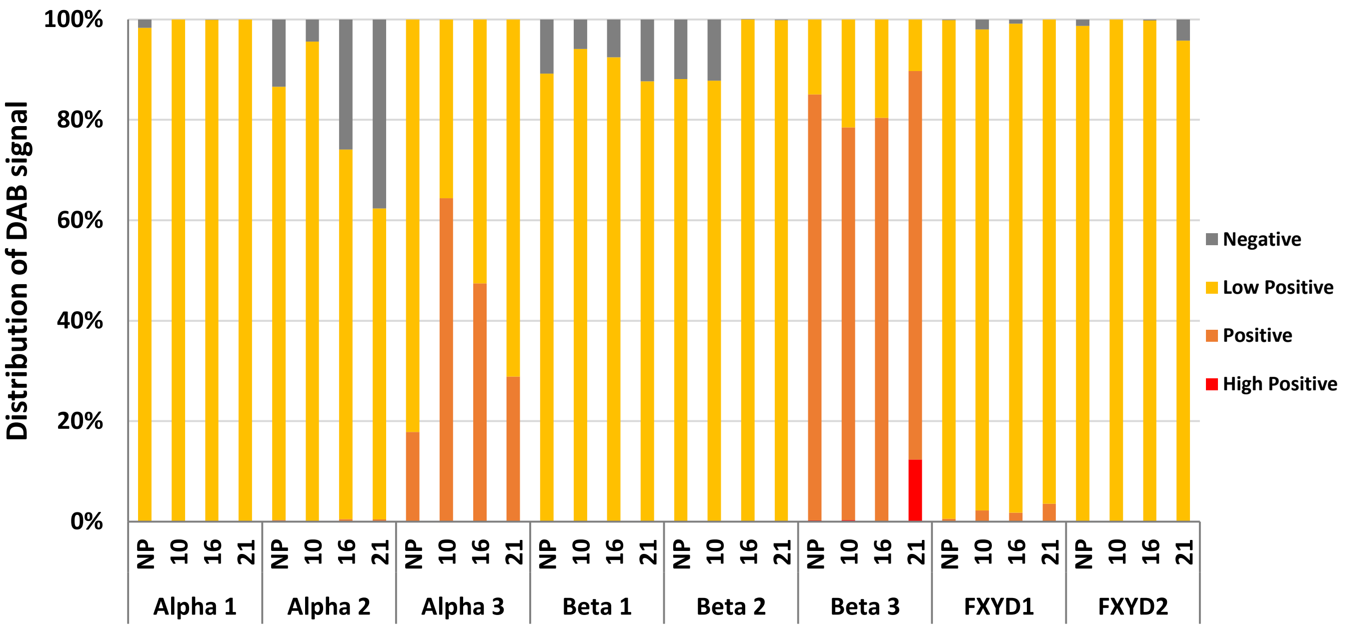

B

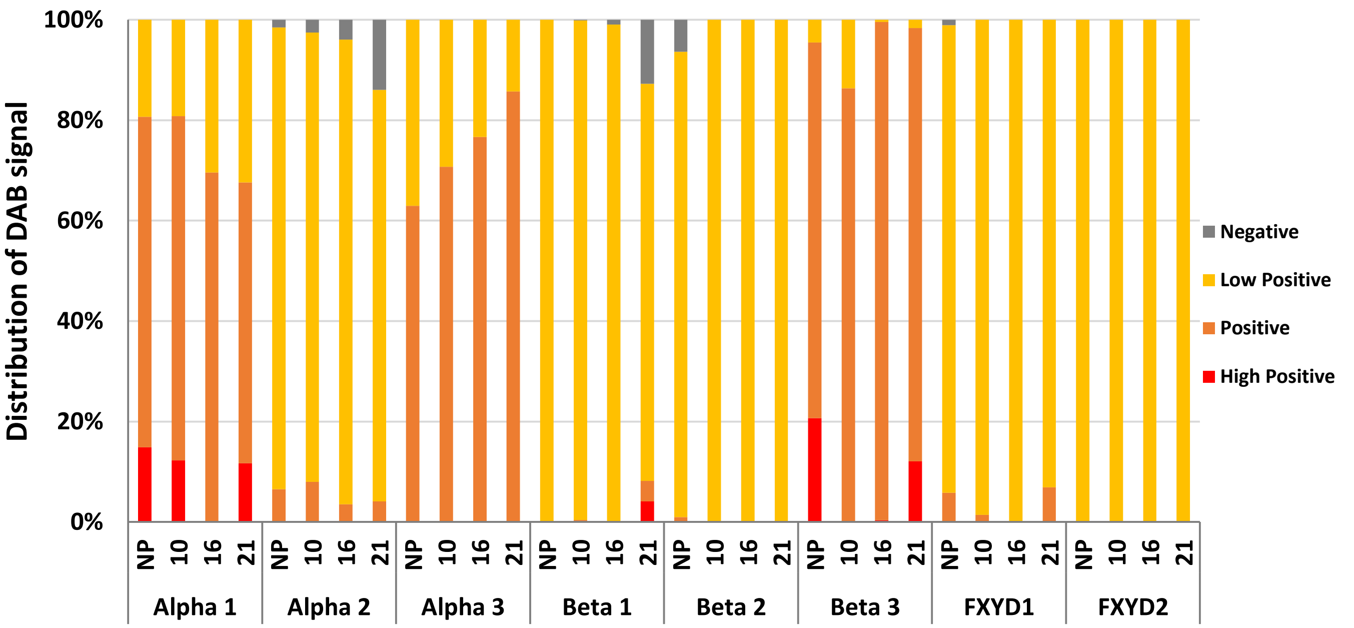

Figure S1 Quantitative evaluation of Na, K ATPase isoform immunoreactivity in (A) smooth muscle and (B) epithelial cell layers using spectral deconvolution
